# The Prevalence and Risk Factors of Gallstone Among Adults in South-East of Iran: A Population-Based Study

**DOI:** 10.5539/gjhs.v8n4p60

**Published:** 2015-07-30

**Authors:** Alireza Ansari-Moghaddam, Alireza Khorram, Mahmodreza Miri-Bonjar, Mahdi Mohammadi, Hossein Ansari

**Affiliations:** 1 Health Promotion Research Center, Zahedan University of Medical Sciences, Zahedan, Iran

**Keywords:** gallstones, prevalence, risk factors

## Abstract

**Objective:**

The present study aimed to evaluate the prevalence and possible risk factors of gallstone disease in the general population.

**Patients and Methods:**

This cross sectional study was carried out on a total of 1522 males and females aged ≥30 years in Zahedan district, South-East of Iran. Data were collected by a validated questionnaire and gallstone diagnosis was assessed by an experienced radiologist using ultrasonography. Logistic regression model was used to identify the association between selected variables and gallstone disease.

**Results:**

The overall prevalence of gallstone in participants was 2.4%. The risk of gallstone was 2.60 times higher in people age 45 and older than those aged 30 - 44 years (Odds Ratio = 2.60, 95% CI; 1.22 - 5.55). Females were 2.73 (95% CI; 1.34 - 5.56) times more likely to have disease compared to males as well. The risk in unmarried individuals was also three times higher than married ones (OR = 2.99: 95% CI 1.02 - 9.16). Additionally, daily physical activity reduced the risk of gallstone disease by 66% (95% CI; 0.18 - 0.86).

**Conclusion:**

In conclusion, increasing age and female gender were risk factors, whereas daily physical activity and marriage identified as protective factors in aetiology of gallstone disease.

## 1. Introduction

Gallstone disease is a major health problem in particularly, in developed world (Acalovschi, 2001). It is also one of the leading causes of digestion-related hospital admissions with high healthcare costs in these countries (Tsai et al., 2004; Henao-Morán et al., 2014). For example, in the UK, about 50,000 cholecystectomies are performing in each year and more than two times of that number, patients are admitting to hospital with gallstone-related episodes (Jazrawi, 2002).

The frequency of gallstone disease varied amongst different countries and ethnic populations as well as various regions of the countries (Khan et al., 2009). In most developed countries, including UK, US, Italy, and the Scandinavian nations, the overall prevalence of gallstone disease was reported between10% to 20% (Hou et al., 2009; Behari & Kapoor, 2012). Additionally, some gallstones studies using ultrasonography reported a prevalence rate of 22% to 54% among these countries (Silva & Wong, 2005). In comparison, the occurrence of disease ranged from 5.2 to 10% in African populations, and 3.1 to 6.1% for populations in Asia (Kratzer et al., 1999). Available evidence comes mainly from patients admitted in hospitals populations suggests an occurrence rate of 0.8% (Zamani et al., 2014) to 6.3% (Farzaneh et al., 2007) in Islamic Republic of Iran as well.

Many previous studies have shown that the gallstone disease risk factors are multifactorial (Panpimanmas & Manmee, 2009). These risk factors include increasing age, sex, dietary, high calorie intake, low fiber intake, high refined carbohydrates, hyper triglyceridaemia, physical inactivity, pregnancy, parity, overweight and obesity (Portincasa et al., 2006; Cariati, 2013; Storti et al, 2005; Panpimanmas & Manmee, 2009; Henao-Morán et al., 2014; Chandran et al., 2014).

For instance, the occurrence of disease increases with age in both genders; (Shaffer, 2005; Behari & Kapoor, 2012; Panpimanmas & Manmee, 2009; Kratzer et al., 1998; Nakeeb et al., 2002) with a high prevalence rate at 50–60 years in both men and women. However, the Gallstones formation are rare in children under the age of 10 years (Jazrawi, 2002; Nakeeb et al., 2002) and adolescents (Nakeeb et al., 2002). Gallstone is also more common among females than males (Pacchioni et al., 2012; Panpimanmas & Manmee, 2009; Moro et al., 2000; Nakeeb et al., 2002; Selvaraju et al. 2010). The risk in females is two times higher than in males (Shaffer, 2005; Panpimanmas & Manmee, 2009), and in the younger adults’ population, the female to male ratio is 1.7 to 4:1 (Pacchioni et al., 2012; Selvaraju et al. 2010). Females had a greater risk of gallstone disease, especially if they had used oral contraception and/or had three or more children (Storti et al., 2005; Moro et al., 2000; Singh et al., 2001; Henao-Morán et al., 2014), or full term pregnancies (Kratzer et al., 1998).

There are some disagreements on the association between triglyceride, cholesterol serum levels, obesity, glucose serum levels and diet with gallstone. The risk of developing gallstone disease was significantly associated with increased TG levels, obesity, duration of obesity, less physical activity, increased glucose serum levels/diabetes, and increased cholesterol serum levels in some studies (Pacchioni et al., 2012; Storti et al., 2005; Pagliarulo et al., 2004; Moro et al., 2000; Zamani et al., 2014). Data also suggests that vegetables and fruits intake are negatively associated with gallstone disease formation while consuming the spicy foods, fried foods and cooking oil increases the risk for gallstone disease. Conversely, a vegetarian or non-vegetarian diet (Chandran et al., 2014; Singh et al., 2001), fruit intake (Chandran et al., 2014) physical inactivity, HDL cholesterol and triglyceride levels and body mass index (Pagliarulo et al., 2004; Singh et al., 2001) were not significantly related to the prevalence of gallstone disease in some documents.

In Iran, the prevalence of gallstone disease and its related risk factors has been little studied on a community basis and most studies were pertaining to patients admitted in hospitals. Therefore, the present study aimed to identify the prevalence and its related risk factors among a sample of general population in South-East of Iran.

## 2. Material Studied

### 2.1 Area Descriptions

#### 2.1.1 Methods and/or Techniques

Using a multistage random sampling method, 50 clusters with an average size of 30 participants aged ≥30 years were selected randomly from the study population in 2012. Firstly, study site-Zahedan District located in South-East of Iran divided into 50 geographical areas. Then, head-clusters (the first selected home/individual as opening point for survey in each area) were determined using random table. After that, trained personnel referred to the first home in every cluster and moved on their right side to cover the entire thirty subjects in every cluster. Having used this approach, 1522 individuals were surveyed.

Interviewers completed a validated questionnaire for each study sample at their residential place by face to face interview and then invited them to attend study center, the day after while fasting. The questionnaire included questions regarding: gender, age, marital status, level of education, employment status, number of pregnancies, daily physical activity, BMI, intake of fruits, vegetables, can, dairy, and fish.

Then, a fasting blood sample collected from each of participants in the morning of the day after interview. At the same place, study participants were given a referral letter to attend a reference hospital in afternoon of the same day for gallstone examination. Consequently, gallstone diagnosis was assessed by an experienced radiologist using ultrasonography. The biochemical parameters such as fasting blood sugar, plasma cholesterol, Triglycerides level, HDL and LDL Cholesterol levels, ALT (SGPT), and Alkaline Phosphatase were evaluated by examinations of mentioned blood samples in an approved laboratory center.

To analyses data, the participants were divided into two age groups (30-44 Years and >45 years). Body mass index (BMI) was classified into two groups of Normal versus overweight and Obese (BMI ≥ 25). The daily physical activities were also defined as Sedentary compared to daily physical activity.

SPSS software (version 17) applied to perform all statistical tests. Logistic regression model was used to identify the association between selected variables and gallstone disease. At first, the association between aforementioned measured variables and gallstone disease were examined by a univariate model. Then all significant variables were included in a multivariate model. A p value < 0.05 was considered statistically significant.

This study was approved by the research ethics committee of Zahedan University of Medical Sciences, Iran. Additionally, verbal, informed consent was obtained from each study subject before participating into the study. Moreover, the names of participants were kept as strictly confidential information and were not to be used in the presentation of results.

## 3. Results

A total of 1,522 participants (52.3% male) were included in the study (Table 1). The age of participants ranged from 30 to 88 years with a mean of 48.05 ± 11.75 years. The mean age of females was slightly higher than that of males (48.8 ± 11.5 vs. 47.3 ± 11.9; P = 0.01). A high proportion of subjects were married (96.6 %). The education level of about 60.5 % of participants was under diploma and remaining had diploma/university education.

**Table 1:** Risk factors associated with Gallstone in a Univariate Logistic Regression Model

Variable	No. (%)	Gallstone	OR (95 % CI)
	Screened	No. (%)	
**Gender**			
Male	796 (52.3)	11 (1.4)	1.00
Female	726 (47.7)	29 (4.0)	2.97 (1.47 - 5.99)
**Age**			
30-44 Years	685 (45)	9 (1.3)	1.00
Over 45 Years	837 (55)	31 (3.7)	2.89 (1.37 - 6.11)
**Marital Status**			
Married	1471 (96.6)	36 (2.4)	1.00
Single, widow/separated	51 (3.4)	4 (7.8)	3.39 (1.16 - 9.91)
			
**Education level**			
Under Diploma	884 (60.5)	32 (3.6)	2.67 (1.22 - 5.83)
Diploma/University Graduates	576 (39.5)	8 (1.4)	1.00
**Employment Status**			
Employee	446 (31.7)	4 (0.9)	1.00
Worker	87 (6.2)	1 (1.1)	1.28 (0.14 - 11.64)
Retired	92 (6.5)	1 (1.1)	1.21 (0.13 - 10.99)
Driver	40 (2.8)	1 (2.5)	2.83 (0.31 - 25.97)
Unemployed	118 (8.4)	5 (4.2)	4.89 (1.29 - 18.50)
Housekeeper	624 (44.3)	26 (4.2)	4.80 (1.66 - 13.86)
**Number of Pregnancies**			
Less than three	962 (66.4)	5 (3.5)	1.00
More than three (Multipare)	486 (33.6)	18 (3.8)	2.25 (1.18 - 4.30)
**Physical Activity**			
Sedentary	1004 (66.4)	34 (3.4)	1.00
Daily physical activity	509 (33.6)	6 (1.2)	0.34 (0.14 - 0.82)
**BMI**			
Obese	900 (59.2)	14 (4.4)	2.07 (1.07 - 4.02)
Normal	620 (40.8)	26 (2.2)	1.00
**Dietary Intake**			
**Can**			
Yes	361 (23.8)	9 (2.5)	1.00
No	1155 (76.2)	31 (2.7)	1.08 (0.51 - 2.29)
**Dairy**			
**Yes**	1310 (86.2)	32 (2.4)	1.00
**No**	209 (13.8)	8 (3.8)	1.59 (0.72 - 3.50)
**Fruits and vegetables**			
Yes	1244 (81.9)	26 (1.2)	1.00
No	275 (18.1)	14 (5.1)	2.51 (1.29 - 4.88)
**Fish**			
Yes	991 (65.3)	17 (1.7)	1.00
No	527 (34.7)	23 (4.4)	2.62 (1.38 - 4.94)

OR= Odds Ratio; CI= Confidence Interval.

The overall prevalence of gallstone in the study participants was 2.4%. It was significantly higher in women (4%) than men (1.4%) with an Odds Ratio = 2.97; 95% CI: 1.47–5.99 (Table 1). There were also significant association between age and frequency of gallstone such that individuals aged > 45 years had considerably higher proportion of gallstone compared than those aged 30-45 years (3.7% and 1.3%, respectively) (Odds Ratio= 2.89: 95% CI; 1.37–6.11). Univariate analysis also suggests significant relationship between marital & employment status; Body Mass Index; physical inactivity; number of pregnancies; fruit, vegetable & fish intakes; fatty liver and SGPT with occurrence of gall stone.

Single, widow, and separated subjects had significantly greater prevalence than married ones (7.8% vs. 2.4%) (Odds Ratio = 3.39: 95% CI; 1.16–9.91). Unemployed and housekeeper individuals were more than 4 times likely to have gallstone disease compared to other job categories. Obese population had significantly more gallstone than individuals with normal BMI (Odds Ratio =2.07: 95% CI; 1.07–4.02). Multiparity also associated with a more than 2-fold higher risk of gallstone compared with the participants less than three pregnancies Odds Ratio = 2.25: 95% CI; 1.18–4.30).

There was an inverse relationship between daily physical activity, intake of fruits, vegetables and fish with risk of gallstone disease formation. Daily physical activity reduced the risk of gallstone by 66% (95% CI: 18%–86%). Similarly, intake of fruits and vegetables decreased the risk of developing gallstone disease by 60% (95% CI: 22%–80%). Fish consumption also diminished risk of disease by 62% (95% CI: 18% - 80%).

Additionally, the following biochemical factors were also identified as significant risk factors for gallstone disease in a univariate model (Table 2): having fatty liver disease (OR = 2.02; 95% CI: 1.07–3.81), abnormal serum HDL cholesterol levels (OR = 1.96; 95% CI: 1.01 - 3.85) and ALT (OR = 4.52; 95% CI: 1.09–18.9).

**Table 2:** Biochemical factors associated with Gallstone in a Univariate Logistic Regression Model

Biochemical parameters	No. (%) Screened	Gallstone No. (%)	OR (95 % CI)
**Fatty Liver Status**			
Normal	903 (59.4)	17 (1.9)	
Fatty Liver	617 (40.6)	33 (3.7)	2.02 (1.07 - 3.81)
**Fasting Blood Sugar**			
Normal	1198 (79.2)	33 (2.8)	1.24 (0.54 - 2.84)
Abnormal	314 (20.8)	7 (2.2)	
**Cholesterol**			
Normal	900 (59.5)	25 (2.8)	1.14 (0.60 - 2.18)
Abnormal	612 (40.5)	15 (2.5)	
**Triglycerides**			
Normal	946 (62.6)	29 (3.1)	1.60 (0.79 - 3.22)
Abnormal	566 (37.4)	11 (1.9)	
**HDL Cholesterol**			
Normal	1189 (78.1)	26 (2.2)	0.51 (0.26 - 0.99)
Abnormal	333 (21.9)	14 (4.2)	
**LDL Cholesterol**			
Normal	569 (37.6)	14 (2.5)	0.89 (0.46 - 1.72)
Abnormal	943 (62.4)	26 (2.8)	
**ALT (SGPT)**			
Normal	1227 (81.2)	38 (3.1)	4.52 (1.09 - 18.9)
Abnormal	285 (18.8)	2 (0.7)	
**Alkaline Phosphatase**			
Normal	13 (0.9)	1 (7.7)	3.12 (0.40 - 24.6)
Abnormal	1499 (99.1)	39 (2.6)	

HDL = High-density lipoprotein; LDL = low-density lipoprotein; ALT= alanine aminotransferase.

Moreover, the present study assessed the effects of independent associated risk factors for GSD by using a multiple logistic regression. Table 3 demonstrated the results of the multivariate logistic regression analysis. After adjustment for confounding variables; age, sex, marital status and physical activity were significantly associated with GSD. The risk of developing gallstone was 2.60 times higher in people age 45 and older than those aged 30–44 years (Odds Ratio = 2.60, 95% CI; 1.22–5.55). Females were 2.73 (95% CI; 1.34–5.56) times more likely to have gallstone compared to males as well. The gallstone formation risk in unmarried patients (Single, divorced, spouse, or widow) was also three times higher than married ones (OR = 2.99: 95% CI 1.02–9.16). Additionally, daily physical activity reduced the risk of gallstone disease by 66% (95% CI; 0.18 - 0.86).

**Table 3:** Risk factors associated with Gallstone Disease in a Multivariate Logistic Regression Analysis

Variable	Gallstone	OR (95% CI)
	No. (%)	
**Gender**		
Male	11 (1.4)	1.00
female	29 (4.0)	2.73 (1.34 – 5.56)
**Age**		
30-44 Years	9 (1.3)	1.00
45 Years and Older	31 (3.7)	2.60 (1.22 – 5.55)
**Marital Status**		
Married	36 (2.4)	1.00
Single, widow/separated	4 (7.8)	2.99 (1.02 – 9.16)
**Physical Activity**		
Sedentary	34 (3.4)	1.00
Daily physical activity	6 (1.2)	0.34 (0.14 – 0.82)

## 4. Discussion

This survey is the first population-based study conducted in South-East of Iran to assess the frequency of gallstone disease. The estimated prevalence rate of gallstone in the present study was 2.4% which is different from the rate reported for Amol City of Iran (0.8%) and the occurrence of gallstone disease at the country level (6.3%) (Zamani et al., 2014; Farzaneh et al. 2007). Moreover, various figures were given for the prevalence of gallstones across the world as well, ranging from 4.1% in Tunisia to 20.8% in New Zealand (United States of America 10%–15%, Bangladesh 5.4%, Germany 7.8%, and 10.7% in Peruvian (Abu-Eshy et al., 2007).

Previous epidemiologic studies suggest a significant relationship between female sex, diabetes mellitus, ageing, race, obesity, hyperlipidaemia, cirrhosis, parity, oral contraceptive use, smoking, and family history of gallstone disease and development of gallstone. In the present study, only female gender, age, marital status and daily physical inactivity remained as significant risk factors in multivariate logistic regression model.

The findings showed a higher prevalence of gallstone disease in population of 45 years and older than people aged 30–44 yrs in the current study. The results were consistent with most of the previous studies come from other parts of the world including Western countries and other parts of Asia, in which older age was identified as a significant risk factor for formation of gallstone disease. The significant relationship between elderly and risk of gallstone development might be possibly explained by Long-term exposure to other related environmental risk factors.

Additionally, there was some evidence that in this population, female had a greater prevalence of gallstone disease than male. Similar results have been reported by previous epidemiologic studies (Murshid, 1998; Pagliarulo, et al., 2004; Panpimanmas & Manmee, 2009; Singh et al., 2001) The higher prevalence of gallstone disease in females than males could be possibly due to sex hormone estrogen differences (Murshid, 1998). Estrogen knows as the primary sex hormone in female. This hormone may increases the cholesterol saturation in bile and it could probably lead to cholesterol gallstone formation (Sharma et al., 2013).

Few studies have reported an association between marital status and gallstone disease formation (Channa & Khand, 2013; Selvaraju et al. 2010) compared to some studies (Jayanthi et al., 1999) which contradict such a relationship. These studies (Channa & Khand, 2013; Sharma et al., 2013) demonstrated that in females, the marriage at earlier age was positively associated with having gallstone diseases, because, the early marriages can lead to have longer fertility periods and higher parity rates. Therefore, the female sex hormones due to fertilities can play excusive role for gallstone formation (Channa & Khand, 2013; Sharma et al., 2013).

However, in the current study, gallstone disease was higher in single, widow, and separated individuals compared to married one. The data showed that in all cases with gallstone were females with the ages of >50 years old multiparity which are known as risk factors of gallstone formation. The presence of a significant effect for marital status in multivariate logistic regression analysis might be due to the correlation with hormonal level as well due to the higher parity rates (Murshid, 1998).

Furthermore, daily physical activity recognized a protective factor for gallstone formation in the current study. Similarly, several studies have revealed an inverse relationship between physical activity and the risk of developing gallstone disease (Storti et al., 2005; Henao-Morán et al., 2009). In comparison, some studies did not find a significant association between gallstone disease and physical inactivity (Pagliarulo et al., 2004). Chuang et al. (2001) found that sport activity but not work and activities in leisure time was inversely associated with gallstone disease. They revealed that the higher levels of percent cholesterol were significantly associated with gallstone disease and they also found that less sport activity was associated with higher levels of biliary cholesterol, percent biliary cholesterol, and serum triglycerides. So, reduction of the biliary cholesterol levels might be one of the many probable mechanisms of physical activities to protect from gallstone formation (Chuang et al., 2001).

This may be possibly happened due to the low plasma high-density lipoprotein (HDL) cholesterol and its important role in the etiology of gallstone disease through independent effect of BMI and body weight on metabolic conditions, and independent effect of physical activity on body weight (Storti et al., 2005; Hou et al., 2009; Henao-Morán et al., 2009). As diabetes mellitus known as gallstone risk factor in some studies (Henao-Morán et al., 2009; Chandran et al., 2014), another mechanism could be the preventive effect of physical activity on insulin resistance and diabetes (Henao-Morán et al., 2009). However, exact mechanism by which the physical activity decreases the risk of gallstone diseases are still unclear (Storti et al., 2005; Hou et al., 2009; Henao-Morán et al., 2009).

One of the study limitations is that data was collected via self-reports by participants. Nevertheless, the researchers strove to ensure the participants about the privacy of information in order to answer the questions correctly. Strengths of the present study were the use of population-based study and by confirming the ultrasound as a diagnostic procedure to determine the gallstones, therefore, may not be underestimate in the prevalence estimation.

In conclusion, the results suggest that increasing age, female gender, daily physical inactivity and marital status are major risk factors for the occurrence of gallstone disease. Accordingly, further investigations are required to elucidate the underlying mechanisms of the sex related differences as well as marital status in aetiology of gallstone disease. Moreover, additional prospective studies are also needed to confirm current identified risk factors and clarify other possible causes for gallstone formation.
